# Label-efficient deep semantic segmentation of intracranial hemorrhages in CT-scans

**DOI:** 10.3389/fnimg.2023.1157565

**Published:** 2023-07-25

**Authors:** Antoine Spahr, Jennifer Ståhle, Chunliang Wang, Magnus Kaijser

**Affiliations:** ^1^Department of Biomedical Engineering and Health Systems, KTH Royal Institute of Technology, Stockholm, Sweden; ^2^Signal Processing Laboratory (LTS5), École Polytechnique Fédérale de Lausanne (EPFL), Lausanne, Switzerland; ^3^CHUV—Lausanne University Hospital, Lausanne, Switzerland; ^4^Department of Clinical Neuroscience, Karolinska Institutet, Solna, Stockholm, Sweden; ^5^Department of Neuroradiology, Karolinska University Hospital, Stockholm, Sweden; ^6^Institute of Environmental Medicine, Karolinska Institutet, Stockholm, Sweden

**Keywords:** computer vision, transfer learning, ICH segmentation, traumatic brain injury, dataset, computed tomography

## Abstract

Intracranial hemorrhage (ICH) is a common finding in traumatic brain injury (TBI) and computed tomography (CT) is considered the gold standard for diagnosis. Automated detection of ICH provides clinical value in diagnostics and in the ability to feed robust quantification measures into future prediction models. Several studies have explored ICH detection and segmentation but the research process is somewhat hindered due to a lack of open large and labeled datasets, making validation and comparison almost impossible. The complexity of the task is further challenged by the heterogeneity of ICH patterns, requiring a large number of labeled data to train robust and reliable models. Consequently, due to the labeling cost, there is a need for label-efficient algorithms that can exploit easily available unlabeled or weakly-labeled data. Our aims for this study were to evaluate whether transfer learning can improve ICH segmentation performance and to compare a variety of transfer learning approaches that harness unlabeled and weakly-labeled data. Three self-supervised and three weakly-supervised transfer learning approaches were explored. To be used in our comparisons, we also manually labeled a dataset of 51 CT scans. We demonstrate that transfer learning improves ICH segmentation performance on both datasets. Unlike most studies on ICH segmentation our work relies exclusively on publicly available datasets, allowing for easy comparison of performances in future studies. To further promote comparison between studies, we also present a new public dataset of ICH-labeled CT scans, Seq-CQ500.

## 1. Introduction

Traumatic brain injury (TBI) is a major cause of death and disability in young adults and the number of people who suffer from TBI each year worldwide is estimated to be 69 million (Dewan et al., 2018). At the event of trauma, mechanical forces of impact can result in intracranial hemorrhage (ICH) which can lead to further brain injury and bleeding progression. Rapid identification of intracranial hemorrhage is therefore crucial in the care of TBI patients. Diagnosis of TBI patients is based on clinical assessment and brain imaging where non-contrast CT is standard, due to its wide availability and low acquisition time. Since TBI is a global burden there is, however, a wide variety of settings of TBI assessment and diagnostics, from resourceful level-1-trauma centers to remote areas with only limited access to radiologists.

Automated and precise detection and segmentation of ICH could add clinical value in providing fast and accurate diagnosis. One challenge in developing accurate segmentation models for ICH consists of its heterogeneity with a wide range of distribution patterns within and around the brain. There are five subtypes of ICH, Intraparenchymal hemorrhage (IPH), Epidural hemorrhage (EPH), Subdural hemorrhage (SDH), Subarachnoidal hemorrhage (SAH), and Intraventricular hemorrhage (IVH), all of which can be represented simultaneously in various slices of a CT head scan. Current classification scores of brain trauma are partly based on these subtypes (Marshall et al., 1991; Maas et al., 2005; Nelson et al., 2010; Raj et al., 2014) however, they are time consuming to use and demonstrate intra- and inter-reader variability. Accordingly, providing robust metrics of hemorrhage subtype distribution could be a step forward in improving prediction models in TBI.

Deep learning algorithms (LeCun et al., 2015) enable efficient processing of high-dimensional input, such as CT images, and show promising performances in the medical domain in a diversity of tasks (Ronneberger et al., 2015; Havaei et al., 2017; Chen et al., 2019b). The task of labeling ICH in CT scans is complex since several intracranial conditions can mimic subtypes of ICH, such as calcification, vascular anomalies, and malignant lesions, among others. Accordingly, an in-depth understanding of neuroanatomy and pathology is needed to discriminate these conditions from one another, something that usually requires a radiologist, an often scarce resource. Given the need of large training datasets, this highlights the need for algorithms using fewer labeled data.

During the past years several studies have explored the use of deep learning algorithms for both automated detection and segmentation of ICH. In one of the first studies, Chilamkurthy et al. (2018) harnessed deep learning for automated detection (classification) of traumatic brain lesions, ICH being one. The following year, Kuo et al. (2019) explored the use of a single neural network to perform both detection (classification) and segmentation of ICH in CT scans. These two studies represent the largest work in the field and present impressive results in large datasets, approximately 300,000 and 4,500 CT scans respectively. In conjunction with additional previous studies the datasets for ICH segmentation are non-public, and until recently there was no benchmark dataset for ICH segmentation, making the comparison of methods nearly impossible. It is thus difficult to gauge whether datasets include a representative sample of ICH. To our knowledge, Hssayeni et al. (2020) presents the only public dataset labeled for ICH segmentation, but it is limited in size and consequently reports lower segmentation performances.

In addition, previous studies focus on fully supervised approaches but recent years' development of semi-supervised models have the potential to further increase the models' performances. The simplest form of semi-supervised learning is transfer learning (Raina et al., 2007; Torrey and Shavlik, 2010) where the model's weights are initialized with those learned using the unlabeled/weakly-labeled data on a pretext task. A few studies have explored semi-supervised methods for ICH segmentation (Wang et al., 2020; Kyung et al., 2022). To the best of our knowledge, only Wang et al. (2020) examined the use of external unlabeled public data, from RSNA (Flanders et al., 2020). Kyung et al. (2022) explored a semi-supervised approach on three private datasets and the public dataset from Hssayeni et al. (2020). Their work follows the same direction as this study as they harness transfer learning, but they explored a single pretext task and they evaluate it mostly on private datasets.

There exists a plethora of generic semi-supervised methods that can be applied in various fields. This study focused on transfer-learning for its simplicity. In this context, self-supervised learning has shown to be effective in learning good features without any labels and a number of generic methods have been reported (e.g., He et al., 2019; Chen et al., 2020b; Grill et al., 2020; Bardes et al., 2021; Lee and Aune, 2021). A subset of state-of-the-art methods was explored.

In the study, we also manually labeled a subset of the dataset for ICH-segmentation and built a supervised baseline as a control and tested whether semi-supervised approaches can improve the performances over this baseline.

In summary, evaluations of label efficient approaches for ICH segmentation are poorly explored and benchmark datasets are lacking. To cope with the aforementioned limitations and to facilitate similar comparisons in the future, we made our labeled subset publicly available.

## 2. Methods

### 2.1. Ethical approval

This study was approved by the Swedish Ethical Review Authority (approval no. 2021-00439).

### 2.2. Datasets

#### 2.2.1. Public segmentation dataset

Recently, Hssayeni et al. (2020) collected and published a public dataset of 75 CT-scans of patients with TBI, among which 36 scans present ICH with the associated segmentation ground truth. The scans were anonymized by blurring the facial components of the skull resulting in non-natural structures around the face. Each scan contains approximately 35 slices, and thus has a small inter-slice resolution of around 5 [mm] where each voxel has the dimension of 0.33 × 0.33 × 5 [mm] [voxel volume = 0.54 mm^3^]. Due to the low inter-slice resolution, 3D algorithms cannot be explored resulting in a reduced scope for the application of the dataset, but it is included in our study for comparison.

#### 2.2.2. CQ500 dataset

In a study on ICH classification, (Chilamkurthy et al., 2018) made a validation set of 491 CT-scans (the CQ500) publicly available, 173 containing ICH. However, the scans are only labeled for classification at the scan level which is not usable in a segmentation task.

#### 2.2.3. RSNA dataset

The Radiological Society of North America (RSNA) has recently released a large public dataset of CT-scans labeled for ICH detection (Flanders et al., 2020). The dataset consists of 752,803 slices from more than 25,000 CT-scans among which 107,933 slices contains an ICH. Each slice is labeled whether it contains an ICH and what types of ICH are present.

#### 2.2.4. Our intracranial hemorrhage labeling of the CQ500 dataset, the Seg-CQ500 dataset

**Figure 1:**
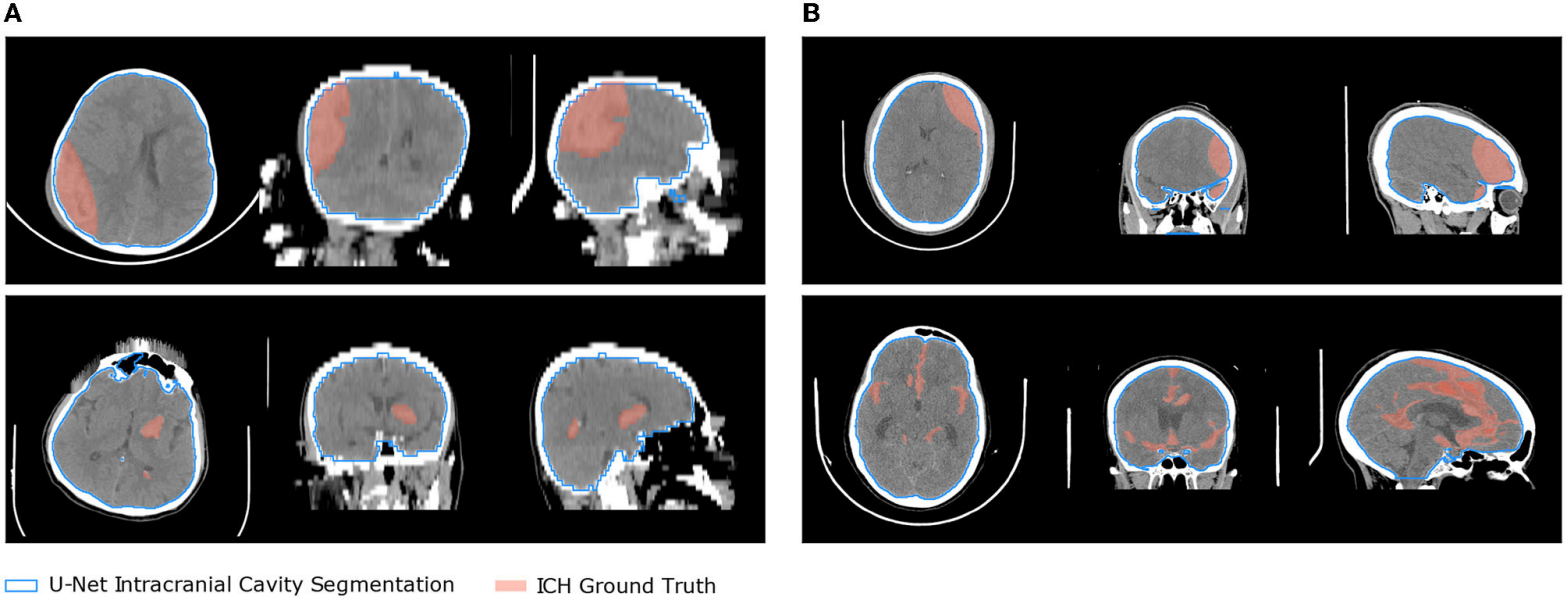
Two samples from the two segmentation datasets are presented overlaid with the ICH ground truth and the intracranial cavity segmentation obtained with the U-Net 2D. **(A)** Hssayeni et al. **(B)** Our Seg-CQ500.

Since the CT scans from the CQ500 dataset are only labeled for classification at the volume level, 51 scans of this dataset were manually labeled in collaboration between a 5th year-radiology resident and an expert neuroradiologist (with over 15yrs experience) from Karolinska University Hospital (Sweden). The scans were randomly chosen ranging from mild to severe injuries and contain all subtypes of ICH along with varying image quality. Mia-lab (Wang et al., 2014) and ITK-SNAP (Yushkevich et al., 2006) software were used for the labeling. Our resulting Seg-CQ500 dataset is versatile with its higher number of scans as well as a higher inter-slice resolution in the majority of the 51 cases. Forty-one cases have voxels with the dimension of 0.45 × 0.45 × 0.625 [mm] [voxel volume = 0.13 mm^3^] and 10 cases have a lower inter-slice resolution ranging from dimensions of 0.48 × 0.48 × 3 mm [voxel volume = 0.69 mm^3^] to 0.49 × 0.49 × 5 mm [voxel volume = 1.22 mm^3^]. As a result, our dataset could be used in future studies for 3D algorithms unlike the dataset of Hssayeni et al. (2020). Samples and information on the Hssayeni et al. (2020) dataset and our segmentation datasets are presented in Figure 1 and Table 1.

**Table 1:** Description of the public ICH segmentation datasets.

	***N***_*****volume*****_								***N***_*****slices*****_		
	**all**	**no-ICH**	**ICH**						**all**	**no-ICH**	**ICH**
			**all**	**IPH**	**IVH**	**SAH**	**SDH**	**EDH**			
Hssayeni et al. (2020)	75	39	36	16	5	7	4	21	2814	2496	318
Our Seg-CQ500	51	0	51	42	12	12	15	5	11175	7675	3500

### 2.3. Pre-processing

#### 2.3.1. Contrast window

The output of a CT scanner is a volume in which each voxel is assigned a physical measure of the light absorption relative to water, given in Hounsfield Units (HU) (DenOtter and Schubert, 2019). As a result, the gray-scale intensity of each voxel is already standardized between samples, but also between scanners. Nonetheless, the voxel values cover a broad range while only a small portion of this spectrum is relevant for ICH segmentation. For example, hematoma usually lies in the range of 40 [HU] to 90 [HU] (Phan et al., 2020). Therefore, in all the following experiments, a window of [−50, 150] [HU] is used to adjust the contrast of the scans in view of focusing on the ICH signature while keeping variety in pixel values to let the networks understand and learn good features. As a result, each scan is rescaled so that values below −50 [HU] are clipped to 0, values above 150 [HU] are clipped to 1, and values inside the window are linearly scaled between 0 and 1. We apply the same contrast window for all the ICH segmentation methods presented below. We keep the pre-processing similar over the methods since the main goal of this study is to compare semi-supervised and supervised approaches.

#### 2.3.2. Data augmentation

To reduce overfitting on the rather limited amount of labeled data for the segmentation task, and in view of learning more robust features, we used data augmentation upon data loading. Since all CT-scans are acquired in the same orientation (head pointing upward and roughly in the center), we decided to use spatial transformations that would yield meaningful and realistic scans. The dataset was thus virtually enriched by translating (vertically and horizontally) the images by a factor uniformly sampled in [−10%, 10%], followed by randomly rotating the images by an angle sampled uniformly in [−15°, 15°], followed by a random scaling of a factor sampled uniformly in [0.9, 1.1], and finally by randomly flipping the images horizontally 50% of the time. Finally, the images were resized to 256 × 256 pixels. Note that different augmentations were used in the contrastive experiment.

#### 2.3.3. Intracranial cavity segmentation

Naturally, ICH can only be present in the intracranial cavity of the patient, and any prediction outside the intracranial cavity would be meaningless. Therefore, we segmented the intracranial cavity for each CT-scan which allowed us to ignore extracranial hemorrhage detection. Motivated by the work of Akkus et al. (2020) we chose to generate the intracranial cavity 3D-mask using a 2D U-Net applied on each slice of the CT-scan. Due to the lack of public data for intracranial cavity segmentation on CT-scans, we decided to manually label 10 scans from the CQ-500 dataset (Chilamkurthy et al., 2018) using the ITK-SNAP software (Yushkevich et al., 2006). In order to obtain a more robust segmentation model for our use-case (with potential presence of ICH), we segmented 5 scans defined as healthy and 5 scans presenting hemorrhage. The 10 scans had good resolution on the z-axis and allowed us to obtain 2,572 labeled images, among which 2106 contained a part of the intracranial cavity. However, due to the large variety in shape and size of ICH, using only 5 scans was considered unlikely to be enough to build a robust model. Therefore, we decided to train two models on different CT windows: 1) One U-Net trained on slices rescaled with a tissue window *HU*∈[0, 600], and 2) One U-Net trained on slices rescaled with a bone window *HU*∈[150, 650]. The model trained on the tissue window should be able to extract the intracranial cavity well in areas where the boundary is less clear, such as at the top and bottom of the skull, while the bone window should perform well when there are abnormal elements in the intracranial cavity, such as ICH, since it is based principally on the skull structure. The final intracranial cavity mask was then obtained as the union of the predictions of those two models.

The tissue and bone U-Nets were trained on the Dice loss (Milletari et al., 2016) for 50 epochs with a batch size of respectively 20 and 16. The weights were optimized using the Adam methods with default parameters and with an initial learning rate of 0.001 exponentially decayed with a base of 0.96 every epoch. The weight of *L*_2_ regularization on the model's parameters was set to 1e-6. The models were trained on the 10 manually labeled scans. Then we segmented the intracranial cavity on the scan of both segmentation datasets. Some results of the segmentation are shown on Figure 1 in blue.

### 2.4. Evaluation metrics

Semantic segmentation is a pixel-wise classification, therefore each segmentation can be viewed as a sample prediction which enables reporting of classification metrics sample-wise (Asgari Taghanaki et al., 2020). To present the performance of the model, we report the mean volume recall which highlights the model's capacity to detect hemorrhage. We also report the mean volume precision which highlights the model's capacity to avoid false detection of hemorrhage. Furthermore we report the mean volume Dice coefficient which is commonly used in segmentation tasks and is equivalent to the F1-score. Thus,


(1) $$\begin{array}{ll} Dice & =\frac{2TP+\epsilon }{2TP+FP+FN+\epsilon } \end{array}$$

(2) $$\begin{array}{ll} Precision & =\frac{TP+\epsilon }{TP+FP+\epsilon } \end{array}$$

(3) $$\begin{array}{ll} Recall & =\frac{TP+\epsilon }{TP+FN+\epsilon } \end{array}$$

where *TP* is the number of true positive, *FP* the number for false positives, *FN* the number of false negatives, and ϵ ensures numerical stability when there are no true positives, set to ϵ = 1. Additionally, these metrics are reported in two ways on the Hssayeni dataset: either including all the 75 volumes, or including only the 36 volumes containing an ICH. The first way allows for taking into account the models' capability to successfully reject healthy scans, but can be biased toward high precision methods. Indeed, a model detecting nothing will be rewarded with maximum performances on healthy volumes which will push the overall performances up while the detection of ICH is null. That is why it is important to also report the performances only on volumes with ICH. As our Seg-CQ500 contains only volumes with ICH, this distinction was not done.

### 2.5. U-Net 2D architecture & baselines

#### 2.5.1. Architecture

The U-Net architecture (Ronneberger et al., 2015) is well suited for semantic segmentation and has shown its potential on various tasks across various domains. Even though the U-Net has been successfully used with 3D volumes as input, such an approach requires the volumes to have relatively similar resolution along the three dimensions in order to learn meaningful convolutional filters. This is not the case in the public dataset of Hssayeni et al. (2020) that have a resolution of around 0.5 mm in the *x* and *y* direction but only around 5 mm between slices. In addition, the use of 3D convolutions is computationally intensive and hinders the possibility of using deeper and more complex architecture. That is why, this study uses a U-Net 2D as the main architecture that processes volumes slice by slice, but the performance was computed over the whole volume instead of separately for each slice. Additionally, it allows harnessing of the large RSNA dataset.

For all experiments, the same U-Net architecture was used for meaningful comparison of the different approaches. Our U-Net was composed of five convolutional blocks for encoding and four deconvolutional blocks for decoding that integrate features of the corresponding block in the encoding path through skip connections. The first convolutional block output a feature map with 32 channels. A convolutional block is a series of two convolutions, batch-normalization and ReLU layers, over which the number of channels is increased by a factor of two (e.g., of feature map dimensions: *h*×*w*×32 → *h*×*w*×32 → *h*×*w*×64). Between the two convolutional blocks a Max-pooling layer reduces the feature map size by a factor of two. A deconvolutional block is composed of a transposed convolution that increases the feature map size followed by a convolutional block similar to the encoding path except that the number of channels is reduced by a factor of two over the block (e.g., of feature map dimensions: *h*×*w*×64 → *h*×*w*×32 → *h*×*w*×32).

#### 2.5.2. Training & evaluation

Given the low number of labeled images in the Hssayeni dataset (*n* = 318) and the heterogeneity of the hemorrhages in the dataset, the performances of the U-Net 2D were estimated through a 10-fold cross-validation scheme to maximize the amount of example available for training. In contrast, on our Seg-CQ500 dataset we adopted a 5-fold cross-validation scheme. The dataset was also split in a stratified way at the level of the volume to ensure that all slices of a scan ended up in the same fold and that each fold contained volumes with hemorrhages. The U-Net was trained on the Dice loss (Milletari et al., 2016) which directly enforces the model to maximize the Dice score:


(4) $$\begin{array}{l} L_{Dice}\left(p,t\right)=1-\frac{2|p\cdot t|}{|p{|}^{2}+|t{|}^{2}} \end{array}$$

where *p* is the output of the network passed through a sigmoid activation, and *t* is the binary ground truth. The binary segmentation was obtained by thresholding the sigmoid output with *t* = 0.5. Note that no class weights were used to compensate the loss for class imbalance.

#### 2.5.3. Baselines

This study focused mainly on transfer learning. To clearly gauge the power of transfer learning, we trained the U-Net 2D without transfer learning by simply training the U-Net 2D using the labeled scans. As a coarse baseline, we first trained the U-Net 2D using only training slices presenting ICH (ICH-only). Second, we hypothesized that the network's optimization should benefit from the presence of healthy slices in the training set as it had to process the whole scan in evaluation. We thus supposed that this additional data would enable the model to learn better features as it would be optimized on a larger variety of data structure helping in discrimination of ICH from other tissues. However, to keep the model's optimization focused on the detection of hemorrhage we decided to assign a smaller weight α to normal slices in the loss. The loss function thus becomes:


(5) $$\begin{array}{l} L_{Dice}\left(p,t\right)=1_{t=0}\;\alpha L_{Dice}\left(p,t\right)+1_{t\neq 0}\;L_{Dice}\left(p,t\right) \end{array}$$

where *1*_*condition*_ is a boolean gate giving 1 if *condition* is true, otherwise 0. We trained the U-Nets on all the ICH train slices together with twice as many normal slices randomly selected in the training volumes, and set α to 0.2 (Mixed).

Both the *ICH-only* and *Mixed* baselines were trained for 100 epochs with a batch size of 16 for the Hssayeni dataset. Since there were more data available in our Seq-CQ500, our baselines were trained for 40 epochs and a batch size of 32. Both were trained with an initial learning rate of 0.001 exponentially decayed at each epoch with a base of 0.96. The model's parameters were optimized using the Adam optimizer with the default parameters and a weight of *L*_2_ regularization set to 1e-6. For both, the generated segmentation were either kept as such or only on the brain using the intracranial cavity mask (ICM).

### 2.6. Transfer learning

**Figure 2:**
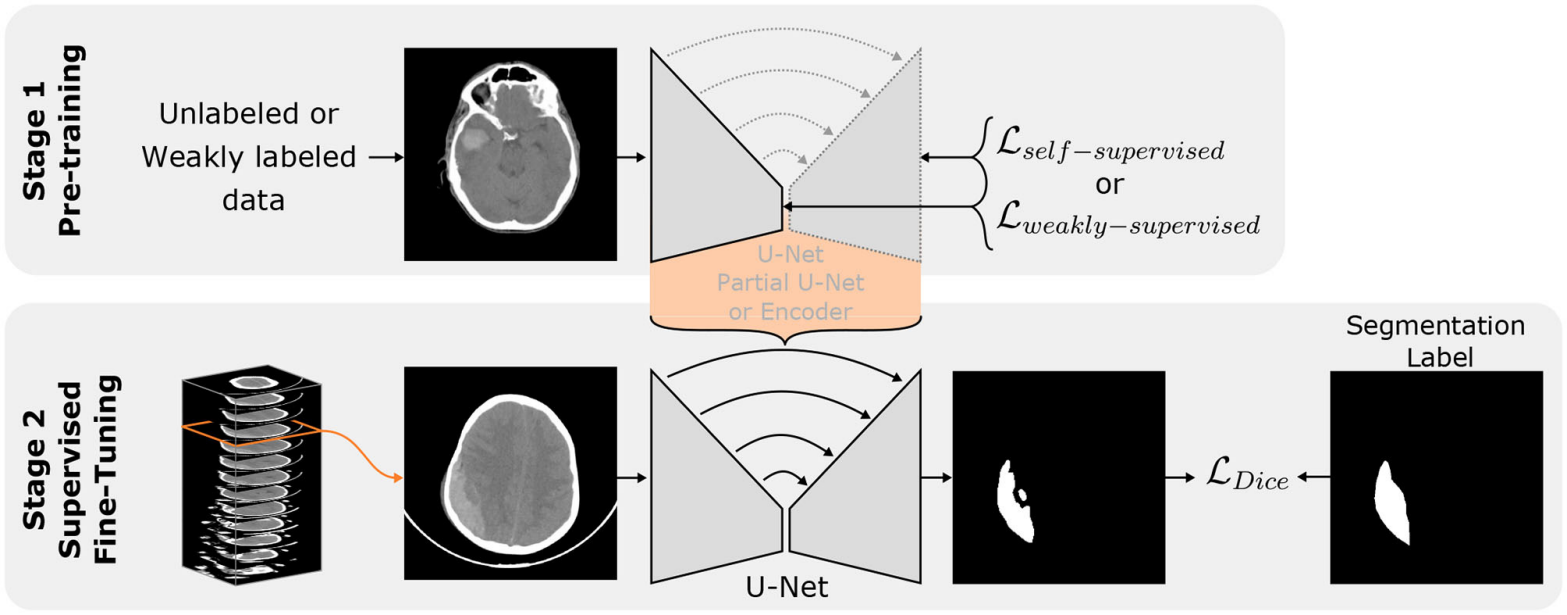
Transfer learning principle. Stage 1 represents the Self/Weakly-supervised pre-train tasks on an arbitrary network architecture (encoder, partial U-Net or full U-Net). The orange bracket highlights the knowledge transfer to a full U-Net. Stage 2 represents the further optimization of the U-Net 2D using available labeled volumes.

In settings where the amount of available annotations is scarce like ours, semi-supervised approaches are effective frameworks enabling the use of unlabeled data to extract meaningful features to assist the model in better performance on the few annotations available. We chose this kind of semi-supervised method because of its simplicity and the absence of cumbersome scaling when dealing with joint training of several tasks. For a fair comparison of the tasks, in all transfer learning experiments we used 107,933 slices of the RSNA dataset labeled with ICH and an equal number of slices without ICH. We kept 5,000 images for validation and used the rest (around 211,000 slices) to train the pretext task. The pre-trained weights were then transferred to the U-Net 2D without freezing them. Afterward, the U-Nets 2D was trained using the few available annotated data similarly to in the *Mixed* setting described above. The general settings are summarized in Figure 2. The label requirements of each approach explored are summarized in Table 2.

**Table 2:** Methods labels requirements overview.

			**Segmentation Label**	**Unlabeled Data**	**Classification Label**	
					**Binary**	**Multi-label**
Semi-supervised	Weakly-supervised Transfer learning	Multi-label classification (DL)	✓	✗	✗	✓
		Multi-label classification (CE)	✓	✗	✗	✓
		Binary Classification	✓	✗	✓	✗
	Self-supervised Transfer learning	Local Contrastive	✓	✓	✗	✗
		Global Contrastive	✓	✓	✗	✗
		Context Restoration	✓	✓	✗	✗
Supervised	No transfer Learning	U-Net 2D Mixed + ICM	✓	✗	✗	✗
		U-Net 2D Mixed	✓	✗	✗	✗
		U-Net 2D ICH-only + ICM	✓	✗	✗	✗
		U-Net 2D ICH-only	✓	✗	✗	✗

#### 2.6.1. Self-supervised transfer learning

Self-supervised objectives aim to learn meaningful convolutional filters using only unlabeled data through a pretext task. The pretext task can be anything that enforces the network to learn features tailored to the data using the data itself as target. The learned filters can then be used to initialize the U-Net or any other compatible networks and be fine-tuned for the task of interest using the few labels available.

##### 2.6.1.1. Context restoration

Chen et al. (2019a) proposed a self-supervised method based on image restoration. For an image *x* from a set of unlabeled data, the rationale of the method is to generate $\hat{x}$, a corrupted version of *x*, by sequentially swapping *N* patches of dimension *h*_*swap*_×*w*_*swap*_ on the image *x*. An encoder-decoder model is then trained to reconstruct *x* from the corrupted version $\hat{x}$ by using the Mean-Square-Error loss with *x* as ground truth. This pretext task is supposed to enforce the network to understand the spatial context of the image and thus learn meaningful features. A U-Net architecture can be used and the learned features can thus be used to initialize the whole model for the segmentation task.

Each image is corrupted with *N* = 20 swaps of dimension *h*_*swap*_×*w*_*swap*_ = 20 × 20 pixels. The network is trained for 50 epochs with a batch-size of 32 using Adam as optimizer with the default parameters. The learning rate is set to 0.001 and is exponentially decayed with a base of 0.96 every epoch. The network's weights are regularized with an *L*_2_-penalty weight of 1e-6.

##### 2.6.1.2. Global contrastive

The contrastive self-supervised task enables to learning of salient features by comparing images in a set and has recently shown promising capabilities in classification (Chen et al., 2020a,c; He et al., 2020) and anomaly detection (Spahr et al., 2021). An encoder network ψ is optimized to bring similar images closer in the embedding space while pushing dissimilar ones away from one another (Oord et al., 2019). To identify similar and dissimilar samples without prior knowledge, an image *x* from a comparison set of images X is heavily augmented twice by the transformation $T_{G}$, resulting in two versions of the same image. The network encoder ψ(·) yields a representation **z** of each image. For each transformed image *x*_*i*_ in the comparison set, the network ψ is trained to identify the corresponding image's (*x*_*j*_) representation from the set of 2*N*−1 other transformed images {_*x*_*k*_}*k*≠*i*_. It can be transcribed into the InfoNCE loss that we will call the global contrastive loss $L_{Global}$ for it processes global representation:


(6) $${ℒ}_{Global}=\sum_{i=0}^{N}-log\frac{exp(sim(z_{i},z_{j})/\tau )}{\sum_{k=1}^{2N}{}_{\left[k\neq i\right]}exp(sim(z_{i},z_{k})/\tau )}$$

where **z**_*i*_ = ψ(*x*_*i*_), *sim*(**u**, **v**) = **u**^T^**v**/||**u**||||**v**|| is the cosine similarity between *u* and *v*, and τ is a hyper-parameter called temperature. It has been demonstrated by Chen et al. (2020a,c), and He et al. (2020) that a large comparison set as well as a strong augmentation yield better learned features for downstream tasks. There are different approaches to define the comparison set: Chen et al. (2020a) use the mini-batch as comparison set while He et al. (2020) use a data bank updated over the training allowing a larger comparison set without the computational cost of a large mini-batch. Chen et al. (2020a) further showed that defining ψ(·) = *MLP*(ψ_*e*_(·)) as the encoder to trained ψ_*e*_(·) yields better performances. The contrastive optimization was therefore performed with an additional MLP projection head, but only the features of ψ_*e*_ were transferred downstream. The features extractor ψ_*e*_(·) can then be used to initialize only the encoder part of the segmentation network. The segmentation model is then trained similarly as in a supervised setting with labeled data.

We defined ψ(·) as the encoder part of the U-Net followed by a 2-layer MLP (512 → 512 → 128). We defined the transformation $T_{G}$ as a sequential combination of random translation in range [−15%, 15%], random rotation in the range [–90°, 90°], random scaling in the range [0.8, 1.2], random horizontal flipping 50% of the time, randomly adjusting the contrast 50% of the time, randomly adjusting the brightness 50% of the time, randomly blurring (Gaussian) 50% of the time, and randomly cropping and resizing the image. The temperature hyper-parameter is set to τ = 0.1. The network was trained for 50 epochs with a batch-size of 60 using Adam as optimizer with the default parameters. The learning rate was set to 0.001 and was exponentially decayed with a base of 0.96 every epoch. The network's weights were regularized with a *L*_2_-penalty weight of 1e-6.

##### 2.6.1.3. Local contrastive

Global contrastive pre-training is designed for learning features of an encoder only making it well suited for tasks involving only an encoder structure such as classification. However, in a segmentation procedure, the network is expected to expand the representation into a segmentation mask. As a result, in the global contrastive approach, around half of the network remains randomly initialized when fine tuning for the segmentation. Chaitanya et al. (2020) recently proposed an adaptation of the contrastive pre-training for the segmentation of organs in CT-scans. In their approach, a contrastive loss was applied locally in the feature map of the decoding path. The core of the idea is that the decoding feature maps of two versions of an image should present local similarities: a region of the feature map should be similar between two versions, but dissimilar to other regions of the feature map. As a result, no comparison is made with other images, only local regions are compared. Formally, for an input image *x*, two augmented versions are obtained by applying the transformation $T_{L}$ twice to obtain *x*_1_ and *x*_2_. Note that we chose $T_{L}$ to not contain transformations that would strongly impair the localization of features (such as horizontal flipping) since the feature localization is the core of the comparison. In order to compare features that were still of a rather low level of abstraction, the comparison was performed on the feature map of an intermediate stage of decoding. As a result, the network ψ(·) was an encoder followed by a partial decoder. The feature map of interest was then passed through a convolutional projection head (series of 1 × 1 convolutions) that have the same purpose as the MLP head in the global contrastive. Therefore the network output **F**_1_ = ψ(*x*_1_) has dimension [*H*×*W*×*C*] in the 2D case. To compare local features, *N*_*r*_ non-overlapping regions of size [*K*×*K*×*C*] are randomly extracted from the feature map obtained for both *x*_1_ and *x*_2_ (**F**_1_ and **F**_2_). It yields 2*N*_*r*_ comparison units **f**_*ij*_ where *i* is the index of image version and *j* is the index of the region. The local contrastive loss can thus be written as:


(7) $${ℒ}_{Local}=\sum_{i=0}^{N_{r}}-log\frac{exp(sim(f_{1i},f_{2i})/\tau )}{\sum_{k=1}^{2N_{r}}{}_{\left[k\neq i\right]}exp(sim(f_{1k},f_{2k})/\tau )}$$

In their study, Chaitanya et al. (2020) first optimized the encoder part of ψ using the global contrastive task, then froze the encoder weight to train only the partial decoding on the local contrastive task. We adopted the same strategy and used the learned encoding and decoding features to partially initialize the U-Net and fine-tune it with the labeled data. Note that skip connections are conserved in the partial network.

We defined ψ(·) as the encoder part of the U-Net followed by three decoding blocks and two 1 × 1 convolution with (64 → 128 → 32). We defined the transformation $T_{L}$ as a sequential combination of random translation in range [−15%, 0.15%], random rotation in the range [–45°, 45°], random scaling in the range [0.8, 1.2], randomly adjusting the contrast 50% of the time, randomly adjusting the brightness 50% of the time, randomly blurring (Gaussian) 50% of the time, and randomly cropping and resizing the image. The temperature hyper-parameter was set to τ = 0.1. We extracted *N*_*r*_ = 20 regions of size *K*×*K* = 3 × 3 from the feature map to compute the local loss. The network was initialized with the weights learned with the global contrastive task (see above) and was then trained for 50 epochs with a batch-size of 24 using Adam as optimizer with the default parameters. The learning rate was set to 0.001 and was exponentially decayed with a base of 0.96 every epoch. The network's weights were regularized with a *L*_2_-penalty weight of 1e-6.

#### 2.6.2. Weakly-supervised transfer learning

Self-supervised tasks are designed to learn relevant features in an unsupervised way to implicitly differentiate ICH from healthy scans. However it can be challenging to encourage the network to focus specifically on smaller ICH, such as widespread traumatic SAH. In contrast, in weakly-supervised transfer learning, with access to weak labels (e.g., classification labels), one can provide an explicit learning signal to discriminate the hemorrhages from the rest.

##### 2.6.2.1. Binary classification

Since the RSNA is labeled for hemorrhages presence at a slice level, convolutional filters of an encoder can be optimized using a binary classification task as the pretext task (ICH vs. no-ICH). We define the classifier as the encoder part of the U-Net followed by an average pooling layer, a 3-layer MLP (512 → 1024 → 256 → 2) and a softmax layer as final activation. The model is then trained for 50 epochs with a batch-size of 64 using Adam as the optimizer with the default parameters, using the cross-entropy loss. The learning rate is set to 0.001 and is exponentially decayed with a base of 0.96 every epoch. The network's weights are regularized with a *L*_2_-penalty weight of 1e-6.

##### 2.6.2.2. Multi-label classification

The RSNA dataset is not only labeled for the binary classification task but also for the classification of ICH subtypes allowing a more complex weakly-supervised pre-training scheme. Each CT slice may contain one, or several, of the five subtypes of ICH. Such a classification task is known as multi-label classification in which the classifier is trained to maximize its sigmoid outputs response for all non-exclusive positives classes through a class-wise binary cross-entropy loss or through a Dice loss. In our case, we chose to use seven different classes: no-ICH, ICH, and the five ICH types. We decided to include the ICH class to train the model explicitly in differentiating non-ICH and ICH slices as in the binary classification task. On top of that, the classification of the five types should push the models in differentiating the nature of the hemorrhage and therefore in learning better features. We used a similar classifier as for the binary classification with seven neurons as output and a sigmoid layer as final activation. We trained the model either on a weighted binary cross-entropy (CE) loss where each possible class is seen as a binary classification task, or on the Dice loss (DL) where the model is trained to generate an output that matches the 7-neurons ground truth. The model was then trained with the same settings as in the binary classification task.

## 3. Results

**Figure 3:**
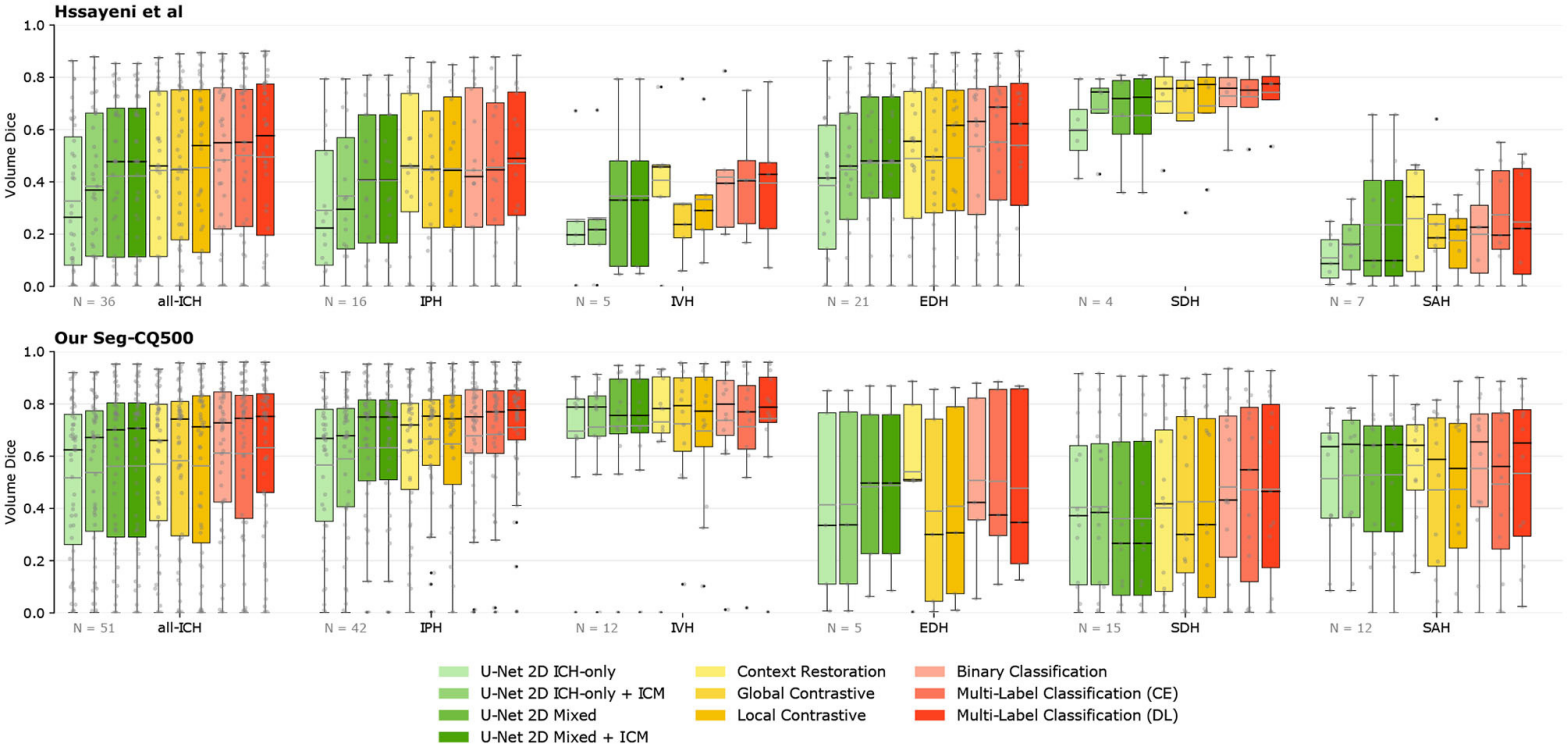
Segmentation performances overview. The volume Dice for each method is presented through boxplots, either for all volumes with ICH or for the different ICH-types [Intraparenchymal (IPH), Intraventricular (IVH), Epidural (EDH), Subdural (SDH), and Subarachnoid (SAH)]. Methods are grouped by color depending on the type of supervision. The further on the right, the more labels are needed. The number of volumes per category is presented on the lower left corner. The gray line on the boxplot highlights the mean while the black highlights the median. ICM, Intracranial Cavity Mask.

All the segmentation performances are presented in Table 3 for both datasets as the mean and standard deviation over all volumes when present in the test fold of the cross-validation. As the dataset of Hssayeni et al. (2020) contains volumes without ICH we also report the performances either considering all the 75 volumes or considering only the volumes with hemorrhages. We make this distinction to avoid possible bias toward methods that do not detect ICH well. Indeed, in a volume without hemorrhage (i.e., without true positives), the Dice, precision, and recall are either 1.0 or decay rapidly toward zero with few false positives. As a result, a method with a low detection rate would see its performance greatly increased when considering the volume without ICH, but it would not reflect the hemorrhage detection capability which is the main objective. The segmentation performances are further visually presented in Figure 3 through the volume Dice distributions to better appreciate and compare the true performances. The distribution is presented over all volumes with ICH but also by ICH sub-type. The boxplot by sub-type must be read with caution for two reasons. First, because the number of volumes with a given type can be low, and second, because a volume may contain more than one sub-type of ICH. Nonetheless, we decided to present them to better grasp the models' capabilities.

**Table 3:** The models' performances are presented over all volumes as the mean Dice, precision and recall (as μ±σ).

	**Hssayeni et al**.						**Our Seg-CQ500**		
	**All Volumes**			**ICH Volumes**			*		
	**Dice [%]**	**Precision [%]**	**Recall [%]**	**Dice [%]**	**Precision [%]**	**Recall [%]**	**Dice [%]**	**Precision [%]**	**Recall [%]**
Multi-label classification (DL)	**63.85**±39.96	**69.33**±38.78	76.45 ± 33.23	49.50 ± 31.95	60.92 ± 33.04	50.95 ± 32.37	**63.23**±29.44	79.77 ± 23.33	59.64 ± 30.74
Multi-label classification (CE)	62.95 ± 39.63	67.31 ± 38.95	**77.03**±32.32	**50.06**±30.77	59.15 ± 32.08	**52.14**±31.35	61.00 ± 30.53	**82.93**±20.73	57.33 ± 31.74
Binary classification	56.00 ± 41.29	61.34 ± 41.20	76.14 ± 33.72	48.32 ± 31.52	59.44 ± 32.99	50.29 ± 32.90	61.19 ± 29.63	78.52 ± 25.16	59.14 ± 31.29
Local contrastive	58.62 ± 41.17	69.31 ± 38.20	73.37 ± 36.02	45.42 ± 32.22	**67.71**±29.66	44.53 ± 33.12	56.27 ± 33.78	79.44 ± 27.62	51.32 ± 33.66
Global contrastive	60.41 ± 40.22	68.48 ± 38.73	73.34 ± 35.56	44.98 ± 29.57	61.78 ± 31.72	44.45 ± 31.99	58.26 ± 31.22	82.50 ± 21.36	53.44 ± 31.87
Context restoration	47.17 ± 41.70	56.36 ± 41.57	72.87 ± 36.71	44.36 ± 31.34	63.51 ± 29.55	43.47 ± 33.75	56.97 ± 29.88	64.77 ± 27.80	**59.76**±30.87
U-Net 2D mixed + ICM	38.39 ± 39.43	46.29 ± 41.56	72.18 ± 36.54	42.22 ± 29.62	58.68 ± 31.09	42.05 ± 32.05	56.22 ± 31.89	69.35 ± 27.68	56.46 ± 32.90
U-Net 2D mixed	38.36 ± 39.43	46.24 ± 41.56	72.18 ± 36.54	42.17 ± 29.60	58.58 ± 31.13	42.04 ± 32.04	56.12 ± 31.95	68.77 ± 28.49	56.46 ± 32.90
U-Net 2D ICH-only + ICM	18.51 ± 27.60	19.22 ± 27.92	73.49 ± 35.85	38.31 ± 28.91	39.78 ± 28.49	44.77 ± 32.96	53.74 ± 30.01	57.13 ± 29.53	59.03 ± 31.94
U-Net 2D ICH-only	15.73 ± 25.22	14.79 ± 24.13	73.49 ± 35.85	32.64 ± 27.92	30.67 ± 27.05	44.77 ± 32.96	51.74 ± 29.78	54.17 ± 29.25	59.04 ± 31.93

The highest mean value is highlighted in bold. For the dataset of Hssayeni et al. (2020) the metrics are reported either over all the volumes or only over the volumes containing ICH. Since our Seg-CQ500 contains only volumes with ICH, this distinction is not done. ICM, Intracranial Cavity Mask.

The straightforward implementation of the U-Net 2D trained only with slices containing ICH yields a rather low mean Dice score over volumes of the Hssayeni dataset (15.73% on all volumes and 32.64% on ICH volumes). This low Dice can be mainly imputed to a low precision of the model that tends to detect hemorrhages everywhere which is not a desired behavior. Subsequently, by simply using the generated intracranial cavity mask to remove insignificant predictions outside of the intracranial cavity, the performances were increased and reached a Dice of 18.51% on all volumes and 38.31% on ICH volumes. Even with this improvement, the precision still remained quite low, reflecting a large number of false positives inside the brain. On the other hand, on our Seq-CQ500 dataset, a mean volume Dice of 51.74% was obtained with a better precision. Again, the use of the intracranial cavity mask further improved the Dice by a few percent.

Interestingly, the simple adaptation of the U-Net 2D training consisting of adding slices without ICH and assigning them a lower weight on the loss computation, easily improved the segmentation capabilities of the U-Net on both datasets by several percent in Dice. The improvement came from a large increase in precision while conserving a rather similar recall. In this case, the application of the intracranial cavity mask does not further increase the scores on both datasets.

The RSNA data was used without the labels in the three different self-supervised transfer learning experiments: context restoration, global contrastive, and local contrastive. Each pre-training scheme improved the downstream segmentation's Dice. On the Hssayeni dataset, the mean volume Dice is improved by 2 to 3% on ICH volume while an increase between 10 and 20% is observed on all volumes. This increase is mostly the result of an improved precision of the U-Net. In our Seg-CQ500 dataset, the Dice is also improved but in a lessened fashion, and a Dice of 58.26% is obtained using the global contrastive task. The two contrastive tasks yielded a large precision but a reduced recall while the context restoration yielded a better recall but a reduced precision.

On both datasets the knowledge transferred from weakly-supervised classification tasks yields higher mean volume Dice compared to the baselines and the self-supervised tasks. On the Hssayeni dataset the best Dice is obtained with the multi-label classification trained on the cross-entropy loss (50.06%), while on our Seg-CQ500 dataset, the best Dice is obtained with the multi-label classification trained on the Dice loss (63.23%). This transfer learning scheme demonstrates a large precision and recall.

**Figure 4:**
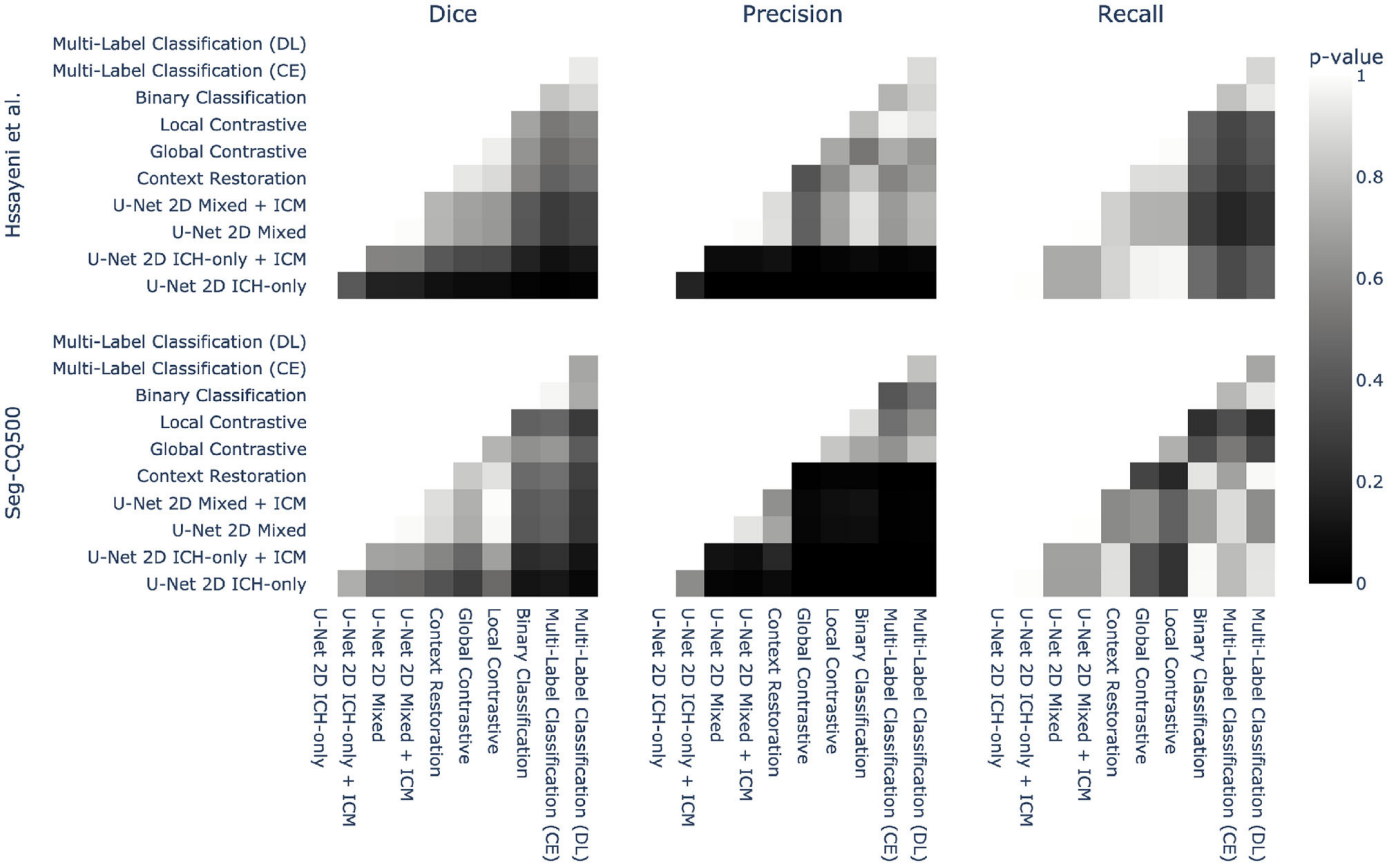
Statistical pairwise comparison of the different methods for both datasets. Each method is compared to the others using a Welch two-sided *t*-test on three different metrics: Dice, precision, and recall. The *p*-value of this test is represented as a heatmap to provide an overview of the statistical significance.

Methods are mainly compared on their averaged metrics over the different volumes. While this provides a general trend, it does not address the statistical significance of the observed improvements. To address this, Welch t-tests were conducted between each pair of methods for each metric and both datasets. Figure 4 presents the corresponding *p*-values for these tests. Regarding recall, the improvement observed between the different methods and the baselines was not found to be statistically significant. However, the precision exhibited a significant improvement with the utilization of semi-supervised methods on both datasets. Consequently, the Dice coefficient did not display a significant improvement due to the modest gains in recall. Nevertheless, a discernible tendency of improvements can still be observed. Overall, these findings demonstrate that semi-supervised models have the potential to significantly enhance precision in the models, while their impact on recall may not be as pronounced.

## 4. Discussion

### 4.1. Benefit of transfer-learning

In the absence of transfer learning, segmentation performances were improved by simply using the intracranial cavity mask to reject several false positives. Such an improvement was not seen in our larger dataset, highlighting the benefit provided by having more data. Furthermore, presenting the model with more images, even without the targeted hemorrhage, greatly improved the performance. We hypothesize that the addition of non-ICH images in the mini-batch allowed the model to better differentiate the healthy structures from ICH structures which successfully reduced the false positives. Noteworthy is that this simple improvement relies solely on the segmentation dataset itself and does not require any external data. It is therefore data-efficient. The absence of improvement using the intracranial cavity mask further highlights that this adaptation allows the U-Net to focus implicitly on the intracranial cavity region.

However, the use of external data in transfer learning allowed higher segmentation performances. In general, among the three self-supervised pre-training schemes, the global and local contrastive tasks appeared to yield better downstream segmentation on both datasets even though fewer weights were transferred to the downstream U-Net. This shows the relevance of the contrastive tasks to learn meaningful features in a self-supervised way. Moreover, the enhanced performance was larger in the smaller (Hssayeni et al., 2020) dataset than on ours, suggesting that with larger datasets the benefit of transfer learning is reduced and having more data act in a similar fashion.

Nonetheless, in the weakly-supervised transfer learning setting, the use of the classification labels allowed explicit learning of what we aimed to learn with the self-supervised: weights that discriminate hemorrhage slices from the other and thus features that are tailored for detecting ICH. Consequently, better performances were obtained using this framework on both datasets showing that even larger datasets can benefit from transfer learning when using more explicit pre-training tasks. In this context, the highest mean volume Dices were thus obtained using the knowledge transferred from the multi-label classification which is also the task that requires the most complex labels. This confirms the ever-existing trade-off between performances and label access.

**Figure 5:**
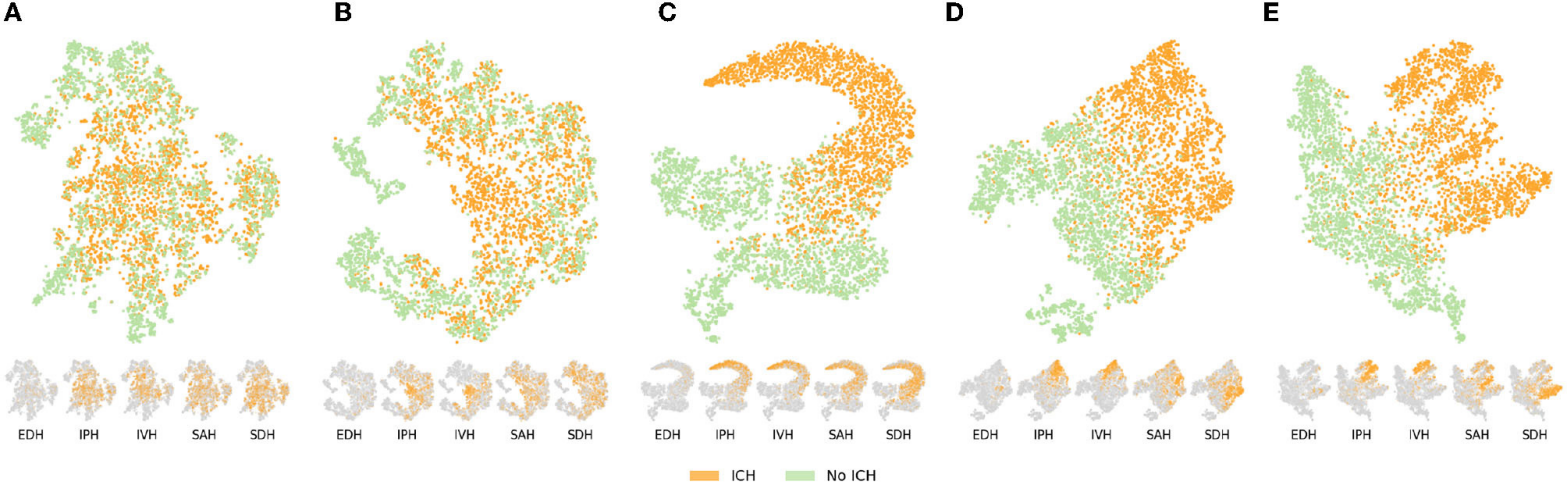
Bottleneck representation learned by the different pre-training tasks (self-supervised or weakly-supervised) on the validation set (*n* = 5,000). The large embedding space is first passed through an average pooling layer and the resulting 512-dimensional vector is represented in 2D thanks to a t-SNE transformation (Maaten and Hinton, 2008). Each sample is colored depending on whether it represents a slice with or without ICH. Below the main visualization, the same t-SNE is presented for the five ICH types in which orange highlights samples of the given type and gray represents the absence of the type. EDH, epidural hemorrhage; IPH, intraparenchymal hemorrhage; IVH, intraventricular hemorrhage; SAH, subarachnoid hemorrhage; SDH, subdural hemorrhage. **(A)** Context restoration. **(B)** Global contrastive learning. **(C)** Binary classification. **(D)** Multi-label classification (CE). **(E)** Multi-label classifcation (DL).

The performances increased compared to the mixed baseline solely rely on the weights used to initialize the U-Net. It is therefore insightful to uncover what those pre-training tasks have learned. That is why we observe the 512-dimensional representation (the feature map transferred to the segmentation model averaged over height and width) of the validation set using a t-SNE transformation (Maaten and Hinton, 2008), colored by ICH presence. The t-SNE visualizations are shown on Figure 5. Note that for a fair comparison, the different models were trained on the same amount of data and evaluated with similar validation sets of 5,000 slices. At first glance, the two self-supervised tasks yield bottleneck representations that look roughly similar: ICH slices tend to be mapped in the same cluster and are slightly blended with some healthy slices, while some non-ICH clusters clearly stick out. Even though the representations look similar, with the contrastive task, there exists an area containing exclusively ICH slices whereas it is not the case with the context restoration task where positive and negative slices are almost always blended. More precisely, the contrastive task seems to isolate intraparenchymal and intraventricular hemorrhages (IPH and IVH) while it is not the case in context restoration. Therefore the self-supervised contrastive method provides the incentive to rely on ICH structures to differentiate images. Being able to rely on those structures highlights the better ability of the contrastive task to learn salient features. In brief, the contrastive tasks have learned features that better discriminate ICH slices from the others. Those better features later transpose in better segmentation performances compared to the context restoration task. Note that the context restoration has the advantage of providing initialized features for the whole U-Net while the contrastive task can only pre-train an encoder. Consequently, with less information transferred, the contrastive task enables better performances.

With the access to classification labels, the representation learned on the binary classification tasks presents a clean separation of ICH and non-ICH slices with a blending area containing challenging cases. As a result, the features learned in this weakly-supervised way are tailored specifically for ICH and naturally lead to better segmentation performances especially in terms of recall. Furthermore, based on the representation by ICH type on Figure 5 it again appears that IPH and IVH are the two types that are well pushed away from healthy slices and thus away from the blending area that characterize challenging cases. Nonetheless, as shown on the t-SNE representation by ICH type, features learned on the binary classification tasks are optimized to differentiate ICH as a whole and the model is not explicitly encouraged to learn more detailed features about those hemorrhages (i.e., what differentiate the ICH types). On the other hand, the pre-training on the multi-label classification task (DL and CE) provides such an incentive to learn higher-level features as demonstrated by the t-SNE representations. Indeed, not only the ICH and non-ICH slices are nicely separated similarly as in the binary classification case, but the different ICH types also form clusters highlighting that the model has successfully learned to discriminate them. The clusters appear to be more separated when using the Dice loss as an objective. Note that the cluster boundaries are fuzzy because of the multi-label settings; one sample can contain ICH of various types.

The subset of our explored methods were considered state-of-the-art at the time of our experiments. We acknowledge that they could potentially be outperformed by other self-supervised methods. Nevertheless, we hope that our results will encourage further exploration and utilization of this vast array of methods in the field of intracranial hemorrhage segmentation and detection.

In brief, the use of unlabeled data through transfer learning enables great improvement of the segmentation of ICH using a relatively simple framework. Additionally the benefit of transfer learning seems to be consistent across the two datasets explored. We further show that better performances are reached when using more specific pre-training tasks using weak labels.

### 4.2. Benefit of our Seq-CQ500

The use of transfer learning allows an increase of the segmentation performances on the two segmentation datasets used in this study highlighting the relevance and robustness of this approach. However the raw performances obtained on the two datasets diverge significantly. Indeed, on the Hssayeni dataset the highest mean volume Dice obtained (only on ICH volumes) reaches 50.06% while on our Seg-CQ500 the highest mean volume Dice of 63.23% is reached. Furthermore, with our Seg-CQ500, the worst mean volume Dice obtained is 51.74%. Our efforts in comparing datasets highlights the large discrepancies that can be observed between different small datasets.

Another example is the discrepancy in Dice between the subtypes IVH and SDH in Figure 3. Here we can see that SDH has a much higher Dice for the Hssayeni dataset than the Seq-CQ500 dataset, while it is the opposite for IVH. This is despite the fact that the Seq-CQ500 dataset has more examples of the subtypes than the Hssayeni dataset. The most likely explanation is that the examples of IVH and SDH differ in heterogeneity and difficulty between the datasets. This example further highlights that large datasets are required to cover the full spectrum of hemorrhages. As a result, it is crucial to use benchmark datasets across studies to clearly gauge the capabilities between research studies otherwise any comparison would be meaningless.

As highlighted in Table 1 our Seq-CQ500 dataset contains more data volumes and more slices compared to the Hssayeni dataset which may explain the overall better results obtained with Seq-CQ500. The evaluation of label-efficient approaches is currently obstructed by insufficient benchmark datasets and even though the size of the Seq-CQ500 limits its comprehensiveness it is more versatile than what is currently publicly available. Our dataset not only contains more labeled scans than the only other publicly available dataset (Hssayeni et al., 2020) but it also provides scans with a better inter-slice resolution (41 out of the 51 volumes). This inter-slice resolution would enable the use of 3D architecture, which is not explored in this study.

Our manual labeling of the Seq-CQ500 dataset was performed by radiologists, something that is always combined with a risk of human errors. The border between ICH and normal brain parenchyma is often indistinct and there is always the risk that factors such as fatigue, concentration, and time of day affect the labeling. Even though this risk is ever present, we were able to differentiate the ICH findings we came across in the CQ500 dataset from ICH mimics and expect the risk of misclassification in our study to be low. We thus believe that our new publicly available Seq-CQ500 dataset can be used as a relevant benchmark dataset in the development of ICH segmentation algorithms.

## 5. Conclusion

In this study, we have demonstrated the relevance of self-supervised and weakly-supervised transfer learning to improve segmentation performance. Additionally, our study on ICH segmentation highlights that the smaller the labeled dataset, the stronger the benefit of transfer learning, whereas larger datasets benefit when using more explicit pre-training tasks. We have also underlined the importance of comparing methods on different datasets. In addition, we present a new open dataset with labels for ICH segmentation, Seq-CQ500 (Chilamkurthy et al., 2018), available as a benchmark for future research in automatic segmentation of ICH.

## Data availability statement

The original contributions presented in the study are publicly available. This data can be found here: https://zenodo.org/record/8063221.

## Ethics statement

The studies involving human participants were reviewed and approved by the Swedish Ethical Review Authority. Written informed consent from the participants' legal guardian/next of kin was not required to participate in this study in accordance with the national legislation and the institutional requirements.

## Author contributions

AS and CW contributed to methodology, data analysis, and experiments. AS wrote the first draft of the manuscript. AS and JS contributed to editing and revision. JS and MK performed manual annotations of CT head. CW and MK contributed to reviewing and supervision. All authors contributed to the article and approved the submitted version.
